# What Are the Biomarkers for Immunotherapy in SCLC?

**DOI:** 10.3390/ijms222011123

**Published:** 2021-10-15

**Authors:** Vito Longo, Annamaria Catino, Michele Montrone, Pamela Pizzutilo, Tiziana Annese, Francesco Pesola, Ilaria Marech, Sandro Cassiano, Domenico Ribatti, Domenico Galetta

**Affiliations:** 1 Medical Thoracic Oncology Unit, IRCCS Istituto Tumori “Giovanni Paolo II”, 70124 Bari, Italy; a.catino@oncologico.bari.it (A.C.); m.montrone@oncologico.bari.it (M.M.); p.pizzutilo@oncologico.bari.it (P.P.); f.pesola@oncologico.bari.it (F.P.); i.marech@oncologico.bari.it (I.M.); sandrocassiano@libero.it (S.C.); galetta@oncologico.bari.it (D.G.); 2 Department of Basic Medical Sciences, Neurosciences, and Sensory Organs, University of Bari Medical School, 70124 Bari, Italy; tiziana.annese@uniba.it (T.A.); domenico.ribatti@uniba.it (D.R.)

**Keywords:** SCLC, biomarkers, programmed death ligand 1, tumour mutational burden, tumour microenvironment, serum anti-neuronal nuclear antibodies, SCLC-I subtype

## Abstract

Small-cell lung cancer (SCLC) is an aggressive malignancy that exhibits a rapid doubling time, a high growth fraction, and the early development of widespread metastases. The addition of immune checkpoint inhibitors to first-line chemotherapy represents the first significant improvement of systemic therapy in several decades. However, in contrast to its effects on non-SCLC, the advantageous effects of immunotherapy addition are modest in SCLC. In particular, only a small number of SCLC patients benefit from immune checkpoint inhibitors. Additionally, biomarkers selection is lacking for SCLC, with clinical trials largely focusing on unselected populations. Here, we review the data concerning the major biomarkers for immunotherapy, namely, programmed death ligand 1 expression and tumour mutational burden. Furthermore, we explore other potential biomarkers, including the role of the immune microenvironment in SCLC, the role of genetic alterations, and the potential links between neurological paraneoplastic syndromes, serum anti-neuronal nuclear antibodies, and outcomes in SCLC patients treated with immunotherapy.

## 1. Introduction

Small-cell lung cancer (SCLC) is an aggressive neuroendocrine malignancy that exhibits a rapid doubling time, a high growth fraction, and the early development of widespread metastases [1]. Despite the addition of immunotherapy to platinum-based frontline chemotherapy, improvements in overall response rate (ORR), progression free survival (PFS), and overall survival (OS) are very low [2,3,4,5]. In this regard, in comparing chemoimmunotherapy and chemotherapy, the divergence of curves after 6 months suggests that only a small proportion of patients with SCLC benefit from immune checkpoint inhibitors (ICIs). In contrast to non-SCLC (NSCLC), where predictive biomarkers of response have dramatically changed treatment approaches [6], biomarkers selection is lacking in terms of SCLC treatment. In fact, clinical trials on SCLC patients are largely focused on unselected populations; therefore, all patients receive standard treatment. Additionally, tissue samples of quantity and quality sufficient to perform a molecular analysis of SCLC are frequently unavailable. Furthermore, the tissue samples of patients with SCLC appear more heterogeneous than expected due to the high biological plasticity of this malignancy and its ability to adapt to different growth conditions. Here, we review the data concerning the major biomarkers for immunotherapy evaluated in lung cancer research, namely, programmed death ligand 1 (PD-L1) expression and tumour mutational burden (TMB). Furthermore, we explore other potential biomarkers, in particular, the role of the immune microenvironment in SCLC, and the potential role of genetic alterations regarding the efficacy of immunotherapy. We also assess the links between neurological paraneoplastic syndromes, serum anti-neuronal nuclear antibodies, and outcomes for SCLC patients, considering the potential role of these antibodies as biomarkers of immunotherapy efficacy and/or toxicity.

## 2. PD-L1 Expression

The expression of PD-L1 in cases of SCLC is reported to be less frequent than in cases of NSCLC. The scarce cellularity of SCLC specimens limits the ability to detect PD-L1 [7]. In spite of previous reports that showed a positive correlation between PD-L1 expression levels with a limited disease (LD) stage and a favourable outcome, currently, the role of PD-L1 expression in SCLC patients is controversial [8]. Only about a third of patients who were enrolled in the IMPower133 trial (137/403) had evaluable tumour tissue. For those patients with adequate quantities of tumour material, the PD-L1 expression level was <1% in tumour cells in almost all cases (129/137), while the PD-L1 expression level in immune cells was <1% in about half of the cases (68/137). No correlations between PD-L1 expression levels in tumour cells or immune cells and clinical outcomes have been found in patients treated with chemotherapy plus atezolizumab (Table 1). Conversely, patients with both PD-L1-negative tumours and immune cells showed an improvement in OS rate (median OS, 10.2 months versus 8.3 months, respectively; HR, 0.51; 95% CI, 0.30–0.89) and PFS rate (median PFS, 5.4 versus 4.2 months; HR, 0.52; 95% CI, 0.31–0.88) receiving chemotherapy plus atezolizumab versus chemotherapy plus placebo. Therefore, these data suggest that PD-L1 expression is not a predictive biomarker in patients with SCLC receiving chemotherapy plus ICIs. An OS benefit was observed in patients with PD-L1 expression ≥5%; however, the number of patients in this subgroup was very low [9]. Concerning the use of atezolizumab in the second-line setting, the IFCT-1603 phase II RCT compared atezolizumab versus chemotherapy. In this study, the tumour PD-L1 expression level was evaluated using the SP-142 assay. Unfortunately, of the 53 evaluable tumour specimens, only one showed tumour PD-L1 expression, excluding the opportunity for evaluations of predictive value [10].

In accordance with the analysis of the IMPower133 trial, the phase III CASPIAN trial, evaluating the use of durvalumab in combination with etoposide plus either cisplatin or carboplatin, showed no significant impact of PD-L1 expression on the effect of treatment. About half of the tumour specimens (277/531) were evaluable, showing low levels of PD-L1 expression. In particular, 22% and 5% of patients had tumours with expression levels ≥1% in immune and tumour cells, respectively. Neither the PD-L1 expression in tumour cells nor in immune cells correlated with better outcomes in patients treated with chemotherapy plus durvalumab [15] Similarly to the CASPIAN and IMPower133 trials, in the phase III study, Keynote 604, the addition of pembrolizumab to a platinum and etoposide regimen was shown to improve the PFS rate (12-month PFS, 13.6% versus 3.1%), and prolonged OS (24-month, 22.5% versus 11%), although the significance threshold was not reached for the OS rate. In this trial, the PFS and OS HRs were shown to be similar in participants with PD-L1-positive as well as PD-L1-negative malignancies [16].

In a single-arm, phase II study investigating first-line maintenance pembrolizumab in patients with SCLC, 66% of the tumour specimens (30/50) were evaluable. Only three patients showed PD-L1 expression levels ≥1%. Among these, one had no measurable disease at study entry and two responded to therapy with a median PFS of 11 months. PD-L1 expression was also detected at the tumour–stromal interface in 8 out of 20 patients who responded or who had stable disease after induction chemotherapy. PD-L1 expression at the tumour–stromal interface resulted in a better outcome, specifically, an improvement of ORRs (37.5% versus 8.3%), PFS (6.5 versus 1.3 months), and median OS (12.8 versus 7.6 months) [11] (Table 1).

The combined positive score (CPS), consisting in the number of PD-L1-positive cells (tumour and immune cells) divided by the total number of viable tumour cells and multiplied by 100, was evaluated in the phase 1b multicohort trial KEYNOTE-028, involving patients with relapsed SCLC who were treated with pembrolizumab. In this trial, a CPS of ≥1% was an inclusion criterion for treatment with pembrolizumab, showing an ORR of 33% (8 of 24) [12]. More recently, KEYNOTE-158, a phase II trial investigating the efficacy of pembrolizumab monotherapy, compared patients with a CPS ≥1% (*n* = 42) with patients characterized by a CPS <1% (*n* = 50). A CPS ≥1% was associated with an improvement of ORRs (35.7% versus 6%) and median OS (14.6 months versus 7.7 months), and an OS rate of 1 year for 53.1% patients with positive CPSs. The ORR was 27% (4 out of 15) among patients with unknown PD-L1 status (Table 1), whereas a pooled analysis of KEYNOTE-028 and KEYNOTE-158, both including patients with PD-L1-positive tumours, showed an ORR of 19.3% (16 out of 83). Concerning responsive patients, there were two complete responses; of these, one concerned a patient with a PD-L1-positive tumour. There were 14 partial responses, with almost all of these regarding PD-L1-positive tumours (13/14). Interestingly, 61% of responders experienced responses that lasted 18 months or longer [12,13].

In a recent single-arm phase II Study, patients with relapsed SCLC were treated with pembrolizumab plus amrubicin until they showed progression. In total, 76% (*n* = 19) of the patients had a CPS ≥1%, showing a better outcome than patients with CPS <1% or those who were not assessable (*n* = 6). In particular, the post hoc analyses demonstrated a higher ORR (58% versus 33%), and mPFS (4.4 months versus 3.0 months, hazard ratio (HR) = 0.73, 95% CI, 0.25 to 1.91). Similarly, patients with higher levels of tumour-infiltrating lymphocytes (TILs) (*n* = 13) had a better ORR and mPFS [14] (Table 1).

Even though PD-L1 seems to be a potential predictive biomarker of response to pembrolizumab in the same population of patients, this was not shown in clinical trials using nivolumab. Concerning the CheckMate032 trial, no differences of outcomes were found between patients with positive PD-L1 expression and patients with PD-L1 <1% or those who were not assessable, both for patients who received nivolumab and for patients who received nivolumab plus ipilimumab [17]. The researchers evaluated tumour PD-L1 expression with a qualitative immunohistochemical assay using Monoclonal Rabbit Anti-PD-L1, namely, 28-8 pharmDx antibody [18]. The tumour specimens were obtained in the pretreatment phase, within 3 months of beginning ICIs therapy. In total, 148 tumour samples were evaluated, and those with ≥100 evaluable tumour cells were considered as acceptable samples. Among patients in the nivolumab monotherapy arm, the ORR was 38% (3 out of 8) if the PD-L1 level was ≥1%, 28% (12 out of 43) if the PD-L1 level was <1%, and 24% (6 out of 25) in those with non-evaluable expression. In the nivolumab (1 mg/kg) and ipilimumab (3 mg/kg) arm, ORRs were 33% (2 out of 6), 36% (8 out of 22), and 33% (6 out of 18), respectively. In comparison, ORRs were 60% (3 out of 5), 24% (7 out of 29), and 15% (2 out of 13), respectively, among these subgroups in the nivolumab (3 mg/kg) and ipilimumab (1 mg/kg) arm [2]. Summarizing the data concerning PD-L1 expression in SCLC, this biomarker does not seem suitable for patients treated with chemotherapy plus ICIs. Additionally, considering the heterogeneity and plasticity of SCLC, future studies should evaluate different biomarkers than PD-L1 expression.

## 3. TMB

As is known, SCLC is often characterized by a high somatic mutation burden due to the association of SCLC with smoking. Hellmann MD et al. evaluated the impact of TMB on the outcome of SCLC patients treated with nivolumab or nivolumab plus ipilimumab in the CheckMate 032 trial. TMB was defined as the total number of somatic mutations, and the patients were subdivided into tertiles using a methodology that was previously reported [19]. The tertiles were defined as: low, <143 mutations; intermediate, 143–247 mutations; and high, ≥248 mutations [17]. Both patients treated with nivolumab and patients treated with nivolumab plus ipilimumab had higher ORRs in the presence of a high TMB level. In particular, patients who received nivolumab monotherapy showed ORRs of 5%, 7%, and 21% in the low (*n* = 42), intermediate (*n* = 44), and high (*n* = 47) TMB tertiles, with a median OS of 3.1 months, 3.9 months, and 5.4 months, respectively. At the same time, the analysis of data from the nivolumab plus ipilimumab group revealed similar results: the ORRs were 22%, 16%, and 46% in the low (*n* = 27), intermediate (*n* = 25), and high (*n* = 26) TMB tertiles, with a median OS of 3.4 months, 3.6 months, and 22 months, respectively (Table 2). Moreover, in a previous study, the same researchers reported no prognostic difference in survival rates based on TMB, suggesting that TMB could be a predictive factor for immunotherapy, rather than a prognostic factor in SCLC patients. Interestingly, whole-exome sequencing for determining TMB correlates well with in silico analysis of a smaller subset of the FoundationOne 315 gene set, assuming the use of the FoundationOne CDx assay as a tool for routine clinical testing of TMB. In the KEYNOTE 158 basket trial, high TMB (≥10 mut/Mb, including 34 patients with SCLC) correlated with better outcomes for all tumour types treated with pembrolizumab, resulting in the FDA’s approval of pembrolizumab in previously treated tumours with high TMB levels. However, caution is needed in interpreting these results due to the over-selection of patients with good prognostic factors enrolled in the phase I/II trials, which explains the longer survival times obtained with anti-PD1 in the third-line setting [20] (Table 2).

That having been said, TMB analysis may not be feasible due to the small size of SCLC biopsies and the necrotic tissue found in SCLC specimens. In this regard, blood (b) TMB was evaluated in SCLC patients treated with first-line atezolizumab plus chemotherapy. The bTMB assay is the same assay used to demonstrate the predictive value of bTMB in patients receiving atezolizumab for the treatment of relapsed NSCLC, obtained by collecting samples from two randomized trials, namely, the POPLARtrial and OAK study [21]. Similar to previous reports, two bTMB cut-offs were used, namely, 10 mut/Mb and 16 mut/Mb. Unfortunately, as already reported in previous studies, these two prespecified cut-offs showed no difference in outcome from the addition of atezolizumab to chemotherapy. In particular, the updated analyses of OS showed a benefit from the addition of atezolizumab in both the low and high bTMB subgroups. In particular, patients with TMBs of <10 mut/Mb exhibited a trend towards improved median OS due to the addition of atezolizumab compared with chemotherapy alone (11.8 months versus 9.2 months, HR, 0.70), whereas patients with bTMBs of ≥10 mut/Mb and of <16 mut/Mb revealed a significant improvement in the median OS by adding atezolizumab to chemotherapy (14.6 months versus 11.2 months, HR 0.68; and 12.5 months versus 9.9 months, HR 0.71, respectively). Finally, patients with a bTMB of ≥16 mut/Mb exhibited a non-significant trend towards improved OS due to chemotherapy plus atezolizumab [9] (Table 2). The significance of these data should be interpreted with caution due to limited patient numbers, particularly in those with a bTMB of ≥16 mut/Mb. Current data suggest that tumour-assessed TMB might have a predictive value in patients with relapsed SCLC treated with immunotherapy. On the other hand, TMB does not have a consistent predictive value in treatment-naïve patients receiving ICIs in combination with chemotherapy, which make up the largest proportion of SCLC patients treated with immunotherapy at present.

## 4. The Role of the Immune Microenvironment

Due to the small number of biopsies with necrotic tissue and the uncommonness of the surgery treatment, studies concerning the immune microenvironment in cases of SCLC are few and retrospective. T cell infiltration in pretreatment biopsies correlates with an improved response to chemotherapy and a better prognosis. However, data concerning the characterization of T cell infiltration are contradictory. The higher expression of CD3, CD20, and CD45, the classic surface biomarkers of TILs, correlates with a better survival rate [22] (Table 3). FOXP3-TILs are usually considered suppressive cells which exert self-tolerance and immune-homeostasis. Their expression often results in an immunosuppressive microenvironment and tumour progression. FOXP3-TILs increase in number from the limited stage to the extensive stage and their expression is also higher in extensive stage (ES)-SCLC with poor prognosis. Nevertheless, FOXP3-TILs are a heterogeneous group, including not only a suppressive population but also a non-suppressive population which exerts anti-tumour activity [23,24]. In this regard, Bonanno et al. [25] reported an independent positive impact of FOXP3-TILs in a cohort including 66 stage I-III patients and 38 metastatic cases. In particular, considering stage I–III patients with FOXP3+ infiltration, they showed a median OS of 52.5 months versus 20.5 months (*p* = 0.027). In agreement with this, in a more recent study including 102 patients with histologically confirmed SCLC at stages I–III, high FOXP3 expression showed longer relapse-free survival times than the low-level group (41.2 months versus 14 months, *p* = 0.008) [26]. On the other hand, in a retrospective study concerning 32 brain metastases from patients with SCLC, FOXP3-TILs were found in 47% of cases, but this result did not have a prognostic impact. The results of these studies are not comparable, however, due to the fact that different sites were examined. The first study performed immunohistochemistry (IHC) mainly on primary tumours, while the second only performed IHC on metastatic samples. Moreover, the analysis of brain metastases revealed a favourable outcome for the presence of CD45RO+ memory T cells and PD-L1+ TILs [27]. The higher expression levels of PD-L1 on TILs resulted in a longer relapse-free survival in the other two studies concerning, respectively, 75 and 102 patients with SCLC, too [28,29]. In contrast, a retrospective series of 205 patients showed a correlation between PD-L1+ and poor prognosis. However, this study only considered resected SCLC patients [22].

Concerning macrophages (MPs), Eerola et al. evaluated 56 surgical samples from patients with SCLC, showing a link between a high number of intratumoural MPs and a favourable outcome. Moreover, intratumoural MPs directly correlated with microvessel density (MD). Nevertheless, the authors did not report the MP/T cell ratio, nor did they report the MP phenotype [30]. In a recent study assessing long-term SCLC survivors, the ratio of CD68-positive MPs to CD3-positive T lymphocytes (CD68/CD3) were lower in long-term survivors (survival >4 years) than in those who survived for the expected amount of time. Moreover, long-term survivors exhibited a higher CD3-positive lymphocyte concentration than patients with the expected survival time (median of 3276 CD3-positive cells/mm^2^ versus a median of 651 cells/mm^2^). In particular, the best discrimination was observed in the tumour stroma zone (*p* = 0.00003); this area revealed the highest number of TILs [31].

Unfortunately, there is a lack of studies examining the tumour microenvironment as a predictive factor for immunotherapy in SCLC patients. A better knowledge of the relationship between tumour microenvironments and immune responses in SCLC patients could promote research into predictive factors for immunotherapy.

## 5. Serum Anti-Neuronal Nuclear Antibodies

Neurologic paraneoplastic syndromes (PNSs) associated with SCLC correlate with a favourable outcome [38,39]. The clinical manifestation of PNSs often precedes cancer-related symptoms, resulting in earlier diagnosis and treatment. However, the superior outcome of SCLC patients with neurologic PNS seems to be linked to other factors. Interestingly, there are several reports that show the spontaneous regression of SCLC without treatment in patients with neurologic PNSs [40,41,42], suggesting that direct immune response against the nervous system may act on neuronal antigens expressed by tumour cells as well [43].

A “hot” tumour microenvironment has been reported in patients with neurologic PNSs. The CD3 levels are higher in SCLC patients with PNSs than in patients with endocrinology PNSs and those without PNSs. Moreover, in SCLC patients with PNSs, a trend toward increased levels of CD4-and CD8-positive T cells has been found, as well as decreased levels of Treg cells and a greater infiltration of activated macrophages [44]. In addition, the analysis of circulating lymphocytes in patients with HuD/anti-neuronal nuclear antibody type 1 (ANNA-1) syndrome revealed an accumulation of HLA DR^+^ CD4 T cells and memory CD45RO^+^CD4^+^ helper T cells. Nevertheless, the mechanisms that explain the better outcomes associated with neurologic PNSs remain unclear. Although neurologic PNSs occur in 3% to 5% of patients with SCLC, about half of all SCLC patients have neuronal antibodies (neur-Abs). Interestingly, SCLC patients with neur-Abs and without neurologic PNSs seem to have favourable outcomes as well. Gozzard et al. [45] evaluated 238 SCLC patients (median OS, 9.5 months), excluding patients with a clinical manifestation of a neurological PNS. In total, 103 patients (43%) had one or more neur-Abs. They found a longer OS in 23 patients (10%) with ANNA-1 antibodies (13.0 months, *p* = 0.037) and in 28 patients (12%) with uncharacterised anti-neuronal nuclear antibodies (ANNA-U) (15.0 months, *p =* 0.0048). No differences have been found with any of the other neur-Abs, including anti-SOX2. Regarding anti-SOX2, two other studies showed that this antibody has diagnostic value in discriminating SCLC-LEMS from non-tumour LEMS but has no relation to survival in patients with SCLC [46,47]. More recently, in a prospective study, Maddison et al. reported that a higher proportion of patients survived longer than 48 months in the group with neur-Abs and neurological PNSs, even if a more limited stage was described in this group [48]. The possible role of neur-Abs as a predictive factor during immunotherapy in SCLC was investigated in a phase II study concerning ipilimumab in combination with carboplatin and etoposide. About 60% (23/38) of patients had at least one positive autoantibody detection. ANNA-1 positivity predicted for a significantly prolonged PFS (10.2 months versus 6.9 months, *p* = 0.032) (Table 3), whereas 3 of 15 patients with positivity for SOX2 presented with ipilimumab-related G3 or higher neurological toxicity [32].

More studies are needed to demonstrate whether neur-Abs are able to be used as biomarkers for immunotherapy efficacy and toxicity.

## 6. Genomic Features

Unlike the increasingly personalized approach to NSCLC, SCLC is still regarded as a single cancer type. SCLC is characterized by clinical and molecular heterogeneity. Firstly, SCLC is classified into neuroendocrine (NE)-high and NE-low subtypes, primarily based on the expression of different neuroendocrine markers, namely chromogranin, synaptophysin, neural cell adhesion molecule 1, and gastrin-releasing peptide [49,50,51,52]. NE-low SCLCs are associated with increased immune cell infiltration; on the other hand, NE-high SCLCs are characterized by low immune cell infiltration, suggesting different responses to ICIs [49].

More recently, Gay et al. [53] identified four SCLC subtypes using the differential expression of transcription factors ASCL1, NEUROD1, and POU2F3 or the low expression of all three transcription factor signatures, accompanied by an inflamed gene signature, namely SCLC-I. The latter showed the greatest benefit from the addition of immunotherapy to chemotherapy. The expression of YAP1 and its transcriptional targets was higher in the SCLC-I group. Using a previously validated EMT score, the SCLC-I group showed in the most mesenchymal subtype [54]. In particular, SCLC-I expressed higher levels of vimentin and AXL and low levels of E-cadherin. Regarding the response to ICIs, the expression of T cell CD8 was shown to be higher in SCLC-I. Moreover, the numbers of other immune cells were increased for SCLC-I, including NK cells, macrophages, and B-lymphocytes. At the same time, SCLC-I expressed higher levels of HLAs and other antigen-presenting machinery. SCLC-I expressed genes including numerous immune checkpoints and human leukocyte antigens (HLAs), as well as PD-L1, PD1, CTLA4, CD38, IDO1, LAG3, and TIGIT. Regarding the latter, a phase III clinical trial examining the addition of tiragolumab, an anti-TIGIT monoclonal antibody, to the carboplatin–etoposide–atezolizumab regimen is ongoing.

A pan-cancer, T cell-inflamed gene-expression profile (GEP) composed of 18 genes indicative of a T cell-activated TME resulted in a correlation with a better outcome in multiple tumour types treated with pembrolizumab [55]. These 18 genes in the T cell-inflamed GEP are identical to the 18 genes in the tumour inflammation signature. Interestingly, T cell-inflamed GEP scores were higher in patients who achieved better ORRs and had longer PFS [33]. Gay et al. observed a higher expression of this GEP in the SCLC-I group of the IMpower133 trial. This trial was not statistically powered for a subtype-specific subgroup analysis. Nevertheless, a subtype-by-subtype analysis comparing survival between chemotherapy plus atezolizumab and chemotherapy alone arms showed a survival benefit across all the subtypes, highlighting that the magnitude of benefit and the median OS with the addition of atezolizumab is higher in SCLC-I. In particular, the addition of atezolizumab in patients with subtype SCLC-I resulted in a median OS of 18 months compared with 10 months without atezolizumab (Table 3). Prospective studies are needed in order to evaluate the role of these subtypes in clinical practice.

## 7. Other Potential Biomarkers

The predictive value of inflammatory cytokines for immunotherapy has been evaluated in SCLC, comparing patients treated with chemotherapy and those treated with chemotherapy plus ipilimumab. The addition of ipilimumab resulted in an increase in various cytokines, including IL-1β, IL-2, IL-4, IL-5, IL-6, IL-8, Mip-1 alpha, GM-CSF, INF-γ, and TNF-alpha. In particular, higher baseline IL-2 levels and an increase in IL-4 levels during immunotherapy correlated with a better OS rate. On the other hand, high TNF-alpha and IL-6 levels predicted resistance to ipilimumab [34] (Table 3).

The lung immune prognostic index (LIPI), calculated from the ratio of derived neutrophils to lymphocytes (dNLR) and lactate dehydrogenase (LDH), was correlated with ICIs outcomes in patients with melanoma as well as NSCLC [56,57,58]. In a multicentric retrospective study concerning 466 NSCLC patients, a pretreatment LIPI, consisting of LDH levels greater than 3 ULN and a dNLR greater than 3, correlated with a poor outcome for ICIs [57]. Recently, a study of 171 patients with SCLC showed the prognostic value of the LIPI, correlating with PFS and OS [35]. Further studies concerning SCLC patients under immunotherapy could explore the role of LIPI and other inflammatory biomarkers (NLR, platelet-to-lymphocyte ratio, etc.) as predictive biomarkers for immunotherapy.

Galectin-9 (Gal-9), a soluble lectin playing a major role in innate and adaptive immunity, was found to be correlated with the PD-L1, PD-1, FOXP3, CD3, CD4, and CD8 levels in SCLC patients. Moreover, higher Gal-9 expression in TILs correlated with better outcomes, suggesting a potential role of Gla-9 as a predictive biomarker in SCLC patients treated with immunotherapy [59].

Human leukocyte antigen (HLA) plays a crucial role in the interaction between the immune system and cancer cells. Recently, a study concerning 102 patients with stage I–III SCLC who underwent radical surgery showed a correlation between HLA class II positivity on TILs and longer recurrence-free survival (40.2 months versus 28.8 months, *p* = 0.014) [36]. At the last WCLC [37], Garassino et al. presented an exploratory analysis of the HLA genotype and survival in the CASPIAN trial. Of the 805 patients enrolled in the study, 414 patients were evaluable for the HLA-I/II genotype. The presence of a particular MHC class II allele, namely, DQB1*03:01, was associated with a longer OS (14.9 versus 10.5 months, HR 0.59) in the durvalumab plus tremelimumab plus chemotherapy arm, but not in the durvalumab plus chemotherapy arm (Table 3).

Further prospective studies concerning these factors linked to inflammation and host immune systems are needed in order to evaluate their predictive properties. On the other hand, due to the high plasticity of SCLC and the low quality of tissue samples obtained in SCLC patients, the study of host-related features could prove useful in the search for biomarkers that predict immunotherapy efficacy.

## 8. Future Perspectives

Despite recent advances in SCLC research, there are still many crucial gaps in our understanding of this disease. The recent addition of ICIs to first-line chemotherapy treatment of ES-SCLC was the first significant improvement of systemic therapy in several decades. However, the magnitude of the treatment effect is very modest [60]. Therefore, the major questions regarding SCLC treatment now are: how can we improve immunotherapy outcomes? and can we identify predictive biomarkers?

As is the case with NSCLC, several studies concerning strategies to improve the efficacy of ICIs are ongoing [61,62]. In particular, research is being carried out to explore the addition of molecules targeting co-inhibitory receptors other than CTLA-4 and PD-1, namely, LAG-3, TIM-3, and TIGIT. On the other hand, studies concerning predictive markers of immunotherapy efficacy are lacking when it comes to SCLC. In this regard, tissue samples from SCLC patients are frequently characterized by low quantity and quality. These features are not only due to intrinsic histological aspects of SCLC samples but also to the absence of clinical practice application for SCLC molecular analysis. This obstacle could be overcome in two ways: firstly, by increasing the quantity of tissue samples; and secondly, by developing blood-based analysis, in particular, analytic methods using circulating tumour cells and blood-plasma-derived exosomes and microvesicles.

## Figures and Tables

**Table 1:** Studies concerning PD-L1 expression in SCLC.

Clinical Trial	Pattern of PD-L1 Expression	ORR	Median PFS	Median OS
Phase II of maintenance Pembrolizumab. [11]	Stromal Interface	ORR 37.5% in 8 patients with PD-L1 positive vs. 8.3% in 12 patients with PD-L1 negative.	Median PFS 6.5 months in 8 patients with PD-L1 positive vs. 1.3 months in 12 patients with PD-L1 negative.	Median OS 12.8 months in 8 patients with PD-L1 positive vs. 7.6 months in 12 patients with PD-L1 negative.
Phase II of maintenance Pembrolizumab. [11]	Tumour cells		Median PFS 11 months among 3 patients with PD-L1 positive.	
Phase III Atezolizumab, carboplatin, and etoposide. [3]	Tumour or immune cells			Median OS 10.2 months in 28 patients with PD-L1 negative in the Atezolizumab arm vs. 8.3 months in 37 patients with PD-L1 negative in the Placebo arm.
Phase Ib of Pembroliuzumab for only PD-L1 positive patients, Keynote-028. [12]	CPS and stroma		Median PFS 1.9 months in 24 patients with PD-L1 positive	Median OS 9.7 months in 24 patients with PD-L1 positive
Phase II of Pembrolizumab Keynote-158. [13]	CPS	ORR 35.7% in 42 patients with PD-L1 positive vs. 6% in 50 patients with PD-L1 negative.	Median PFS 2.1 months in 42 patients with PD-L1 positive vs. 1.9 months in 50 patents with PD-L1 negative.	Median OS 14.6 months in 42 patients with PD-L1 positive vs. 7.7 months in 50 patients with PD-L1 negative.
Phase II Study, patients with relapsed SCLC treated with pembrolizumab plus amrubicin. [14]	CPS	ORR 58% in 19 patients with PD-L1 positive vs. 33% in 6 patients with PD-L1 negative or not assessable.	Median PFS 4.4 months in 19 patients with PD-L1 positive vs. 3.0 months in 6 patients with PD-L1 negative or not assessable.	

CPS, combined positive score; PD-L1, programmed death ligand 1; OS, overall survival; ORR, overall response rate; PFS, progression free survival.

**Table 2:** Studies concerning TMB in SCLC.

Clinical Trial		Type of TMB	ORR	Median PFS	Median OS
Phase III Ateolizumab, carboplatin, and etoposide. [3]		Blood-based TMB			Different blood-based TMB subgroups exhibit similar benefit from addition of atezolizumab to chemotherapy.
CheckMate 032. [17]	Nivolumabmonotherapy arm.	Tissue TMB	ORR 21.3% in patients in the highest TMB tertile receving nivolumab vs. 6.8% and 4.8% in the medium and low tertiles, respectively.	Median PFS 1.4, 1. 3, and 1.3 months, in the high, medium and low TMB tertiles in response to nivolumab, respectively.	Median OS 5.4, 3.9, and 3.1 months, in the high, medium, and low TMB tertiles in response to nivolumab, respectively.
	Nivolumab plus Ipilimumab arm.		ORR 46.2% in patients in the highest TMB tertile receving nivolumab plus ipilimumab vs. 16% and 22% in the medium and low tertiles, respectively.	Medium PFS 7.8, 1.3 and 1.5 months in the high, medium and lowTMB tertiles in response to nivolumab plus ipilimumab, respectively.	Median OS 22, 3.6, and 3.4 months in the high, medium, and low tertiles in response to nivolumab plus ipilimumab, respectively.
Phase II of Pembrolizumab Keynote-158. [13]		Tissue TMB	ORR 29.4% in 34 patients with TMB-high vs. 9.5% in 42 patients with Non-TMB-high status.		

TMB, tumour mutational burden; OS, overall survival; ORR, overall response rate; PFS, progression free survival.

**Table 3:** Other potential biomarkers.

Potential Biomarkers	Summary	References
Immune microenvironment	High concentration of TILs correlates with better outcome and limited stage.	[22,23,24,25,26,27,28,29,30,31]
Serum anti-neuronal nuclear antibodies	Prolonged PFS (10.2 months vs. 6.9 months, *p* = 0.032) has been reported in a phase II study concerning ipilimumab in combination with carboplatin and etoposide, for patients with ANNA-1 positivity.	[32]
Genomic features	A SCLC subtype, characterized by an inflamed gene signature namely SCLC-I, showed a trend toward a better OS (18.2 vs. 10.4 months) by the addition of atezolizumab to chemotherapy.	[33]
Cytokines	Higher baseline IL-2 levels and increase in IL-4 levels during immunotherapy correlated with better OS. Whereas, high TNF-α and IL-6 levels predicted resistence to ipilimumab.	[34]
LIPI	LIPI showed a prognostic value for SCLC patients. No data concerning a potential predictive value of LIPI are available for SCLC patients.	[35]
HLA	A particular MHC class II allele, namely DQB1*03:01, has been associated to a longer OS (14.9 vs. 10.5 months, HR 0.59) in the durvalumab+tremelimumab+chemotherapy arm of the CASPIAN trial.	[36,37]

ANNA-1, anti-neuronal nuclear antibodies 1; Human leukocyte antigen, HLA; LIPI, lung immune prognostic index; OS, overall survival; ORR, overall response rate; PFS, progression free survival.
